# Chemical Communication in Insects: New Advances in Integrated Pest Management Strategies

**DOI:** 10.3390/insects14100799

**Published:** 2023-10-03

**Authors:** Angel Guerrero, Gadi V. P. Reddy

**Affiliations:** 1 Department of Biological Chemistry, Institute of Advanced Chemistry of Catalonia—CSIC, 08034 Barcelona, Spain; 2 Southern Insect Management Research Unit, United States Department of Agriculture (USDA)—Agricultural Research Service (ARS), Stoneville, MS 38776, USA; gadi.reddy@usda.gov

Chemical communication plays a pivotal role in many insect behaviors, including food-seeking, recruitment, the recognition of congeners, reproduction, alarm, territorial marking, and survival. Many of these behaviors are regulated by semiochemicals, which are chemicals able to induce inter- and intra-specific chemical communication.

Semiochemicals have great potential for use in integrated pest management (IPM) programs; pheromones can be particularly effective because they are species-specific, display low acute toxicities to mammals and other beneficial organisms, are active at extremely low doses, and are environmentally benign, leaving no harmful residues behind. In the last few decades, there has been a huge considerable volume of literature dealing with the successful application of pheromones combined with other management strategies in IPM programs, such as the monitoring of pest populations, mass trapping, mating disruption, attract-and-kill, and push–pull programs (for review see f.i. [1]).

In this Special Issue, a number of articles aim to highlight recent new advances in IPM strategies, such as the discovery of novel sex and aggregation pheromones; benefits associated with pheromone-baited traps in monitoring and mass trapping; simultaneous monitoring for several different pests with food-baited lures; and mating disruption experiments to manage forest pest populations. In addition, a review dealing with the development of attractive targeted sugar baits (ATSBs) to suppress outdoor biting mosquito populations and the discovery of new attractants for Brachyceran flies using generic noctuid lures is also presented.

Brassicaceae are essential components of human diets in many countries. In 2020, cabbage accounted for a harvested area of ca. 3,395,300 ha, yielding 105,069,400 tons [2]. The diamondback moth (DBM) *Plutella xylostella* (L.) is a significant factor in the economics of Brassicaceae cropping, and it has been estimated that the annual worldwide cost to manage this pest is USD 4–5 billion. Following previous works implemented in 2020, González and coworkers demonstrate [3] that mass trapping using pheromone-baited traps in Costa Rican and Nicaraguan cabbage plantings led to a significant reduction in insecticide applications, generally increasing the yields, savings, and profits of the farmers. Overall, the yields of Costa Rican farms increased with concomitant lower insecticide costs and an increase in net profit of USD 1723/ha. In Nicaraguan farms, the average increase in net profit was USD 605/ha, essentially due to a dramatic reduction in insecticide application (one-third of the current farmers’ practices). With these results in hand, farmers with previous high insecticide application rates against DBM were the most receptive to the idea of using pheromone-baited traps and reduced their regular practice of calendarized insecticide applications. The authors’ results reinforced previous reports on the control of DBM in India, in which significantly lower numbers of DBM larvae and percentages of plants with holes in leaves within plots treated with pheromone-baited traps were noticed compared to control sites in which regular calendarized insecticide applications were made [4].

The pistachio nut (*Pistacia vera* L.) is one of the most popular tree nuts in the world and is valued globally for its nutritional value and economic importance. The global production of pistachios has increased dramatically over the past few decades, from around 50,000 tons in 1970, to 500,000 tons produced globally in 2000, to more than one million tons in 2020 [5], with the United States (47%) being the major producer country, followed by Turkey (30%) and Iran (19%). Recently, a comprehensive study conducted in Spain by Gómez and coworkers [6] highlighted the leaf beetle *Labidostomis lusitanica* (Germar) (Coleoptera: Chrysomelidae) as a potential serious threat to pistachio production in the Iberian Peninsula, since it has been estimated that the insect may defoliate young trees in a few hours. In their paper, López and coworkers [7] provided the first evidence of a biologically active male-specific compound that may promote male and female aggregation in the field. Males collected from aggregates of both sexes, commonly found in pistachio leaves, release a sex-specific compound, which was identified, using solid-phase microextraction (SPME) followed by gas chromatography–mass spectrometry, as 2-isobutyl-3-methoxypyrazine. The chemical elicits a strong electrophysiological response, with females displaying a higher response than males overall. In addition, males and females responded positively to the compound when they were released individually in a double-choice olfactometer. Although 2-alkyl-methoxypyrazines may act as allomones, aggregation, alarm, or trail pheromones in insects of different orders ([7] and references cited therein), no pyrazine has been identified as a pheromone in leaf beetles so far. Future work in the field is required to ascertain the role of the compound in the leaf beetle’s chemical ecology.

Cutworms and armyworms (Lepidoptera: Noctuidae) are part of a pest complex that may cause economic damage to annual field crops in North America [8]. In Canadian Prairie agroecosystems, the bertha armyworm (*Mamestra configurata* Walker); the redbacked cutworm, *Euxoa ochrogaster* (Guenée); and the pale western cutworm, *Agrotis orthogonia* Morrison are the most common species with localized outbreaks in canola and cereal crops. Female-produced sex pheromones of most cutworm and armyworm species were used in monitoring programs in the 1980s, but these programs were not endorsed since the moth trap catches did not correctly reflect crop damage, likely because moths were attracted to the traps from long distances. Moreover, pheromone-based monitoring programs require individual traps and specific lures for each species, making monitoring several pests costly and time time-consuming. Batallas and Evenden [9] explored the development of a food-based semiochemical lure to monitor both sexes of the cutworm and armyworm pest complex with a single trap and lure and with a minimum impact on native pollinators. Field experiments were conducted in canola and wheat fields in the Canadian Prairies to evaluate the activity of the AAMB lure (mixture of acetic acid and 3-methyl-1-butanol). This lure is a previously developed [10] food bait based on microbial volatile compounds from by-products of fermented sugar baits, to be used alone or in combination with 2-methyl-1-propanol or phenylacetaldehyde in comparison to the respective sex pheromone. As expected, the food bait lures caught lower numbers of targeted moths compared to sex pheromone-baited traps, but both males and females of multiple cutworm species were attracted to the AAMB lure. The low number of moths captured in the food bait traps suggests that only moths from close areas can detect and are attracted to the food bait lures. Future studies should estimate the attractive radius of these lures and determine whether trap catches accurately represent the local population density of the target species.

The pine-tree lappet moth, *Dendrolimus pini* L., is a harmful defoliator of pines in Europe and Asia and a potential invasive species in North America [11]. Large outbreaks of the pest have been registered in history, such as the outbreak in Poland in 2011–2014, the largest one in the previous 70 years that covered about 184,000 ha. Trees highly defoliated by the pest become more susceptible to secondary pests and environmental stress and, therefore, the early detection of increasing populations of insects is crucial for outbreak prevention. The sex pheromone of *D. pini* was identified as a mixture of (*Z*5,*E*7)-dodecadienal ((*Z*5,*E*7)-12:Ald) and (*Z*5,*E*7)-dodecadien-1-ol ((*Z*5,*E*7)-12:OH) [12], but traps baited with the aldehyde alone or mixtures with the alcohol were clearly ineffective in catching a sufficient number of insects (less than one male per trap and day) [13]. Recently, Rudzinski and coworkers [14] identified (*Z*5)-12:Ald, (*Z*5)-12:OH, (*Z*5)-10:OAc, and (*Z*5)-14:OAc as new likely components of the pheromone emitted by females, in addition to the previously discovered (*Z*5,*E*7)-12:Ald and (*Z*5,*E*7)-12:OH. The mixture of all components, except (*Z*5)-10:OAc, which has a repellent effect, resulted in an effective lure for *D. pini* males and provided a basis for further optimization of the lure composition for monitoring *D. pini* populations. The addition of Scots pine essential oil enhanced the performance of the lures [15].

Mating disruption (MD) is considered the most developed pheromone-based technology for the direct management of insect pests and invasive species [1]. The species-specificity and low toxicity of the pheromone applications have led researchers to consider MD as a reliable tool to control insect pests, particularly in areas of low population densities. The spruce budworm *Choristoneura fumiferana* Clemens (Lepidoptera: Tortricidae) (SBW) is a major defoliator of balsam fir and spruce *Picea* spp. in North America. SBW outbreaks occur periodically every 30 to 40 years and may last 15 years in a particular location. From the 1970s, numerous MD experiments for SBW management have been implemented and significant advances in methods and application technologies have taken place [16]. Roscoe and coworkers [17] recently tested a new sprayable microencapsulated formulation (CONFOUND_SBW_), specifically designed for low-density SBW populations (<7 L2/branch) in New Brunswick, Canada. The formulation only contained a mixture of (*E*11)-tetradecenal (95*E*:5*Z*) and (*Z*11)-tetradecenal without any new recently identified secondary pheromone components(*Z*)-11-hexadecenal, (*Z*)-5-tricosene, and (*Z*,*Z*,*Z*)-3,6,9-tricosatriene [18]. The product was designed to adhere to branches after application, thus remaining in the tree canopy where most SBW matings occur. Therefore, the likelihood of exposure to the synthetic pheromone was increased. However, although there was a significant trap catch reduction of 90% in treated blocks compared to untreated control blocks, population densities following treatment were not significantly affected when compared to densities in control blocks. It has been suggested that trap shutdown may need to exceed 95% of population reduction for a successful MD experiment [19]. The lack of population reduction after treatment indicates that the application of CONFOUND_SBW_ at a rate of 50 g of active ingredient per ha is ineffective in controlling SBW populations. Considering that the application cost of CONFOUND_SBW_ is ca. 4× greater than currently registered insecticides used for SBW protection, and the possible inclusion of the other identified secondary components (see above) would further increase the price, the replacement of insecticide use by a pheromone formulation in MD trials appears to be challenging.

Mosquito control is presently the most effective strategy for mosquito-borne diseases, which cause more than 700,000 deaths annually [20]. Pesticide applications have been the backbone of mosquito control programs, but the effectiveness of these interventions continues to decline due to the rapid spread of insecticide resistance and non-target effects in addition to public and environmental safety concerns. To address these concerns, increased efforts to explore additional integrated management strategies have emerged, including approaches that involve the behavioral management of vectors. Attractive targeted sugar baits (ATSBs) are a vector control approach that manipulate and exploit the sugar-feeding behavior of mosquitoes to deploy insecticides. The ATSB technology consists of an attractive compound to the target vector, a sugar component that promotes feeding (feeding stimulant), and an oral insecticide to induce mosquito mortality/morbidity after ingestion of the solution [21]. ATSBs can reduce mosquito densities and clinical malaria incidence when used in conjunction with existing vector control strategies. In their review, Njoroge and coworkers [22] revised the available literature regarding the utility of ATSBs for mosquito control, providing an overview of ATSB active ingredients (toxicants), attractants, modes of deployment, target organisms, and the potential for integrating ATSBs with existing vector control interventions. Particularly noteworthy is their revision of the RNA interference (RNAi) technology as a useful research tool that could potentially be applied in operational vector control strategies [23]. In addition to the use of ATSB insecticides targeting adult mosquitoes, attempts to simultaneously kill immature stages of *Aedes* and *Culex* mosquitoes, among others, using ATSBs formulated with biopesticides [24] have also been successful in the laboratory, but more efforts should be made in field studies in the future.

During field tests implemented in West Ukraine in 2015, plant volatile traps designed to catch Lepidoptera pests caught a large number of flies as non-target insects [25]. Traps were baited with two types of generic lures originally developed for noctuid moths and based on fermenting liquid and floral compounds. The attractants included a semisynthetic bisexual lure (SBL) containing isoamyl alcohol (3-methyl-1-butanol, frequently occurring in fermenting molasses) + acetic acid + red wine in a 1:1:1 ratio, and synthetic floral lures (FLO) containing a 1:1:1 mixture of phenylacetaldehyde, eugenol and benzyl acetate, and a 1:1 blend of phenylacetaldehyde and (*E*)-anethol. The number of specimens identified at the family level amounted to 6501 from 26 families, with the most abundant families represented by more than 500 individuals being Muscidae, Ulididae, Sarcophagidae, and Calliphoridae. A total of 14 of the 26 sampled families were represented with more than 30 individuals/trap. Among the lures assayed, the ternary mixture of the SBL was the most efficient. Thus, SBL traps attracted significantly more specimens of the Muscidae, Ulididae, Sarcophagidae Calliphoridae, Sciomyzidae, Drosohilidae, Phoridae, and Platystomatidae families than FLO and unbaited control traps, while FLO traps were more efficient in catching flies of the Empididae and Milichiidae families. The SBL lure is the first reported attractant of the Heleomyzidae and Sciomyzidae families, and its attractivity to moths has been reported in a wide range of Geometridae, Thiatiridae, and Erebidae species and Noctuidae, particularly in the Noctuinae, Xyleninae, and Hadeninae subfamilies ([25] and references cited therein). Synthetic and semi-synthetic generic noctuid lures could serve as a basis for further studies related to the monitoring and management of new fly pests, vectors, and parasites.

In summary, we hope that this SI highlights the importance of continued research into chemical communication in insects, particularly toward integrating this information into IPM programs. Advances in current technologies and the development of new ones will be of great usefulness in future sustainable pest management programs.
